# The Dictionary of Foods and Drinks

**Published:** 1856-04

**Authors:** 


### THE DICTIONARY OF FOODS AND DRINKS;

### A COMPENDIUM OF ALIMENTARY SUBSTANCES IN USE THROUGHOUT THE WORLD: THEIR HISTORIES, DIETETIC PROPERTIES, AND ADULTERATIONS.

### PART IV.

ALKANET (*Anchusa tinctoria*), a plant of the natural order, *Boraginaceæ*. It is used to give a yellow colour to some foods, as butter. See *Alkanet,* under *Adulterants*.

ALLSPICE. A West-Indian spice, the fruit of the *Eugenia pimento,* natural order *Caryophyllaceæ*. It is an aromatic, and is used commonly to give a flavour to foods. Owing to its cheapness, this spice is not commonly adulterated. But it is used as an adulterant in France in the formation of the spurious pepper-corns, called Lyons pepper. Allspice is a harmless substance as an article of food. Allspice owes its aroma to a volatile oil, which may be distilled from the berries, and is sometimes used in medicine.

ALLIGATOR PEAR, the fruit of the *Persea gratissima*. The tree is a native of the West Indies. The pear often weighs upwards of a pound. The part eaten, and which is a delicious food, is a buff butyraceous substance, lying beneath the rind. It has nutritious properties.

ALMOND, the fruit of the *Amygdalus communis,* natural order *Rosaceæ*. The tree grows in the south of Europe, in Spain, Barbary, Syria, Sicily and Greece. There are two varieties of almond fruit, bitter and sweet. The sweet almond is largest in size, and sweet; the bitter almond is smaller and bitter, and gives off an odour, communicating a peculiar sensation at the back of the throat. This taste is due to a specific principle in the bitter almond, called amygdalin. From this substance an essential oil is obtained, which is sold in the shops as oil of bitter almonds, and which ordinarily contains from 8 to 14 per cent. of prussic acid. The flavour bestowed on articles of food by the bitter almond, or its oil, is due in most part to the acid above named; although, according to Dr. D. Maclagan, it is sometimes free from this deleterious substance. As a medicine, the bitter almond is most useful when properly administered; but its use for dietetic purposes, and by ignorant persons, is most reprehensible, since dangerous effects must and do occasionally arise from its employment; moreover, the bitter almond, as an article of diet, is quite unnecessary, and is only used for the absurd purpose of gratifying the taste

The sweet almond furnishes an useful oil, which is not dangerous; and the fruit itself is eaten at dessert, and is pleasant and wholesome. The almond should always be blanched before being eaten, as the husk sometimes gives rise to intestinal irritation and eruption on the skin. When the sweet almond is bleached, pounded, and mixed with water, an emulsion is formed, which is useful as an adjunct to some medicines. Oil of sweet almonds has been considered nutritive, and has laxative properties. It is, however, not much used, either as an article of diet or as a medicine.

The sweet and bitter almonds, with their oils, were known as foods to the ancients, under the name of the "Greek or Thasian nuts." It was believed that they conferred on those who took them the power of drinking immoderately; and the followers of Apicius mixed together almonds pounded, honey, eggs, oil, milk, garum, and pepper, and made thus a dish which was considered a great delicacy.

ALOE. The broken leaves of the aloe are prepared and used occasionally in the adulteration of tea. See *Adulterants*, art. *Aloe.* The properties of the aloe are medicinal only.

ALUM (*Sulphate of Alumina and Potass*). A crystalline salt, found naturally in Italy, in alum shale. Alum is largely used as an adulterant. See *Adulterants*, art. *Alum.*

AMBROSIA (*α* not, and *βροτος* mortal). The food of the gods, supposed to convey immortality on all those who ate of it; hence the name. It flowed, says the fable, from the horns of the goat Amalthæa, and was nine times sweeter than honey.

AMANITA, the Siberian intoxicating fungus (*Amanita muscaria*), a vegetable of the natural order *Fungaceæ.* This fungus resembles the common mushroom, and is a native of Kamschatka. It is collected, according to Professor Johnstone and other authorities, in the hot months, and is dried, or is left to ripen and dry. When steeped in the expressed juice of the whortleberry, the fungus gives up a principle which possesses intoxicating properties. But a common practice is, to roll the fungus up into a bolus, and thus swallow it. One or two fungi, thus taken, will produce an intoxicating effect which will last from six to twelve hours. If water be taken at the same time, the symptoms are very much increased. The physiological action of the fungus is remarkable: in some persons it acts like laughing-gas; sometimes it causes spasmodic attacks. Another peculiarity is, that the intoxicating agent passes off freely by the kidneys, and imparts to the renal secretion intoxicating powers; and it is a fact supported by the testimony of all who have visited Kamschatka, that the renal secretion, thus acted on, is taken as a stimulating and intoxicating beverage.

AMARANTHUS, a plant of the natural order *Amarantaceæ*. Some species of amaranthus are used as spinach. They are insipid (*Lindley*). The amaranth flowers in autumn, and grows in the south of England.

AMILOT, a Mexican white fish. This fish is a choice article of diet; it measures a foot in length. It is caught in rivers and lakes.

AMMODYTE, the sand-eel, an animal of the class *Pisces*, order *Malacopterygii apoda* (Cuvier). The sand-eel is caught by digging into the sand at low water. It is common on the sandy shores of Britain, and forms a delicious article of food.

AMYLUM. The technical term for starch. See *Starch*.

ANCHOVY (*Engraulis*), class *Pisces*, order *Malacopterygii abdominales* (Cuvier). A fish common in the Mediterranean and on the coasts of Portugal, Spain, and France. It is also said to have been taken on the coasts of Britain. Its length is from four to five inches. The fishermen of the Mediterranean remove the heads and entrails of these fishes, wash them in salt water, and pack them with salt. The fishermen of Provençe colour the salt with ochre, and do not change the brine. The fishermen of the north use only bay-salt, and change the brine several times, which renders their anchovies less acrid. (Soyer.) Anchovies, preserved in one or other of these ways, are imported under the name of Gorgona anchovies, Sicilian, Dutch, etc. They are brought to this country in barrels, and are bottled after importation. Anchovies are nutritious and pleasant articles of diet. In their native regions they are eaten fried, but in this country only in their preserved state. They are commonly used in salads, or to flavour sauces, etc. Sometimes they are fried in paste, sometimes eaten with toasted bread. Anchovies are subject to adulterations. An inferior species of anchovy, having no flavour, is imported from Holland. The anchovy is frequently coloured—commonly after being brought into this country—with Venetian red and Armenian bole, the former of which sometimes contains lead; hence the fish should always be well washed before it is eaten.

ANCHOVY PASTE. A potted meat, prepared from anchovies. It is a pleasant, and when pure, a wholesome article of food. It is frequently adulterated, both with inferior species of anchovy and sometimes with other kinds of fish, and even ordinary meats. It is commonly coloured with Venetian red and Armenian bole. The natural colour of the paste is a pale pink; and its flavour in this pure state is best. Flour, chalk and plaster of Paris are also adulterations.

Anchovy Pear. A fruit common to Jamaica, used by the natives as an article of food.

Anchovy Sauce. A condiment prepared by pounding anchovies in a mortar, warming them with a small quantity of water, adding Cayenne pepper, and straining through a hair sieve. The sauce is adulterated for the sake of colour with Venetian red or Armenian bole, both of which impair the flavour, and are otherwise objectionable.

ANGELICA, the garden angelica, a plant of the natural order *Umbelliferæ*. It grows in England in watery places, flowering from June to September. It is used occasionally as a herb in broths, as a vegetable flavouring substance. The root and fruit have been used in medicine.

ANISE, the *Pimpinella anisum*, a plant of the natural order *Umbelliferæ*. The seed of the plant is used in foods and also as medicine, to give an aromatic flavour. In food the seed is generally added to cakes. The seed yields about three per cent. of a volatile transparent oil, upon which the sweetness and aroma depend. Hemlock seed has sometimes been sold by mistake for anise-seed, and fatal results have followed: the peculiar odour of the anise is its best test. *Anise-seed* is a harmless and pleasant adjunct to foods, *Anise-oil* is an agreeable aromatic; both are used to flavour liquors, as aquardiente, aniseed gin, and the like.

APPLE, the fruit of the *Pyrus Malus*, a tree of the natural order *Rosaceæ*, sub-order *Pomeæ*. Apples are mentioned at an early period of history, as forming an article of food in the East and in Greece; and the tree was carefully cultivated by the Romans, who are said to have introduced it into France. It is also said, with much probability, that the apple is a native of Britain; its aboriginal form being the wild crab-apple, a small, dry, sour, unpalatable fruit. By a careful system of propagation, aided no doubt by a blending with the varieties originally brought from the East and from Southern Europe, some thousands of varieties have been produced, all of which are more or less nutritious and wholesome. The subjoined information regarding this well-known fruit is supplied by Mr. J. H. Tucker.

The importance of apples as food has not hitherto been sufficiently estimated nor understood in this country. Besides contributing a large quantity of sugar, mucilage, albumen, and other nutritious matter, they contain a combination of vegetable acids, extractive matter, and aromatic principles, with the nutritive matter, as to act powerfully as refrigerants, tonics, and antiseptics ; and, when freely used in times of scarcity by rural labourers and others, they prevent debility, strengthen digestion, correct the putrefaction of nitrogenous food, avert scurvy, and probably maintain and strengthen the powers of productive labour. The operatives of Cornwall consider apples nearly as nourishing as bread, and more so than potatoes. In scarcity, apples, instead of being converted into cider, have been in that county sold to the poor ; and the labourers have asserted that they could work on boiled apples without meat, whereas a potatoe diet required either meat or fish. The French and Germans use apples extensively ; indeed, it is rare that they sit down in the rural districts without having them in some shape, even at the best tables. The labourers and mechanics depend on them to a very great extent, and frequently dine on sliced apples with bread. In some other countries, however, apples are less appreciated ; in Belgium, the use of them was forbidden by the authorities on the advent of cholera in 1854.

Apples are used as food in various forms. For the most part, those in which the amount of acid is moderate, while the saccharine or aromatic principles predominate, are eaten in the raw state. The unripe fruit, or those containing a large quantity of acid, as well as some of the milder tasted varieties, are formed into various articles of cookery, by being stewed, roasted, or made into puddings, etc. Mr. Tucker informs us, that "stewed with rice, red cabbage, carrots, or by themselves, with a little sugar and milk, they make a pleasant and nutritious dish." Apples have been given as food to cows, with the effect of causing them to produce a more abundant and richer milk than they had hitherto done. The apple is largely cultivated in England, principally in the counties of Somerset, Devon, and Hereford, and also in France and North America, for the purpose of obtaining from it the well known beverage termed cider. For this purpose the more acid varieties are chiefly employed. In Normandy, we learn from Professor Johnstone, not fewer than five thousand varieties are cultivated for this purpose ; and it is remarked, that the apples there produce a cider of different flavour, according as they grow on chalk, sandy, or clay soils.

